# miRNAs and Müller Glia Reprogramming During Retina Regeneration

**DOI:** 10.3389/fcell.2020.632632

**Published:** 2021-01-18

**Authors:** Gregory J. Konar, Claire Ferguson, Zachary Flickinger, Matthew R. Kent, James G. Patton

**Affiliations:** Department of Biological Sciences, Vanderbilt University, Nashville, TN, United States

**Keywords:** miRNA, Müller glia, retina, regeneration, zebrafish

## Abstract

The use of model systems that are capable of robust, spontaneous retina regeneration has allowed for the identification of genetic pathways and components that are required for retina regeneration. Complemented by mouse models in which retina regeneration can be induced after forced expression of key factors, altered chromatin accessibility, or inhibition of kinase/signaling cascades, a clearer picture of the key regulatory events that control retina regeneration is emerging. In all cases, Müller glia (MG) serve as an adult retinal stem cell that must be reprogrammed to allow for regeneration, with the end goal being to understand why regenerative pathways are blocked in mammals, but spontaneous in other vertebrates such as zebrafish. miRNAs have emerged as key gene regulatory molecules that control both development and regeneration in vertebrates. Here, we focus on a small subset of miRNAs that control MG reprogramming during retina regeneration and have the potential to serve as therapeutic targets for treatment of visual disorders and damage.

## Introduction

In mammals and humans, the extent of spontaneous repair after retina injury or disease is either non-existent or extremely limited (Karl and Reh, 2010). Rather than regenerate, damaged mammalian retinas commonly undergo reactive gliosis and scar formation (Bringmann et al., 2006). This lack of a complete regenerative response to damage directly limits the treatment options for retinal based diseases such as age-related macular degeneration or Stargardt's disease (Link and Collery, 2015; Zarbin, 2016). Numerous strategies are currently being tested to address this limitation, including gene therapy approaches and transplantation of stem cell-derived progenitor cells (MacLaren et al., 2006; Pearson et al., 2012; Cehajic-Kapetanovic et al., 2015; Roska and Sahel, 2018; Stern et al., 2018). An attractive alternative strategy for treatment is to induce endogenous MG-derived regeneration of the retina as is observed in fish and amphibians (Hamon et al., 2016; Lahne et al., 2020). Zebrafish have the ability to regenerate a large array of tissues and organs (Gemberling et al., 2013). One goal for these studies is to determine the factors and pathways that allow for persistent and spontaneous regeneration. Focusing on the retina, knowledge gained from zebrafish studies (Wan and Goldman, 2016; Yao et al., 2018; Hoang et al., 2020; VandenBosch et al., 2020; Zhou et al., 2020) can be applied to identify common mechanisms that induce mammalian retina regeneration. Here, we will focus on the explicit role of miRNAs during MG reprogramming.

## Retina Regeneration in Zebrafish

The retina forms from the central nervous system (CNS) and develops into a three-layered structure consisting of seven main types of cells and numerous other cell types identified by single cell RNAseq (Macosko et al., 2015). The structure, function, cell types, and genes expressed in the retina are largely conserved among vertebrates, supporting the notion that information gained from models capable of spontaneous regeneration might apply to mammals whose regenerative capacity isn't clear (Hitchcock and Raymond, 2004; Stenkamp, 2007; Hamon et al., 2016).

The three main layers that constitute the retina include the outer nuclear layer (ONL), inner nuclear layer (INL), and ganglion cell layer (GCL). The INL and ONL are separated by a thin synaptic layer called the outer plexiform layer (OPL), and the INL and GCL are separated by a thick synaptic layer called the inner plexiform layer (IPL). The ONL contains rod and cone photoreceptors. The INL contains three types of interneurons: bipolar cells (BCs), horizontal cells (HCs), and amacrine cells (ACs). Ganglion cells (GCs) populate the GCL, collect information from BCs, and send signals to the brain for higher order visual processing. In addition to these neuronal cell types, Müller glia (MG) constitute the main glial cell type spanning all three layers of the retina.

## MÜLLER Glia-Derived Regeneration

The unique behavior and placement of MG following damage led to hypotheses that they play an integral role in retina regeneration. Multiple lines of evidence, largely from zebrafish, strongly support MG as the source of retina progenitors after damage. First, new retinal progenitor cells (RPCs) formed in the INL migrate along MG processes to the ONL (Raymond and Rivlin, 1987; Vihtelic and Hyde, 2000; Wu et al., 2001; Raymond et al., 2006). Second, MG become mitotic after damage (Braisted et al., 1994; Vihtelic and Hyde, 2000; Wu et al., 2001; Faillace et al., 2002; Yurco and Cameron, 2005; Raymond et al., 2006). Third, gene expression profiles of MG and RPCs are very similar (Hoang et al., 2020). Most directly, the Raymond lab showed that zebrafish MG produce rod precursors during development and also produce RPCs that can differentiate into any retina cell type following damage (Bernardos et al., 2007). The mechanism by which MG produce RPCs is by dedifferentiation of the MG, owing to the fact that shortly after damage, zebrafish MG begin to produce markers of neural progenitors such as Pax6, α-tubulin, and BLBP (Fausett and Goldman, 2006; Raymond et al., 2006; Thummel et al., 2010).

**Figure 1:**
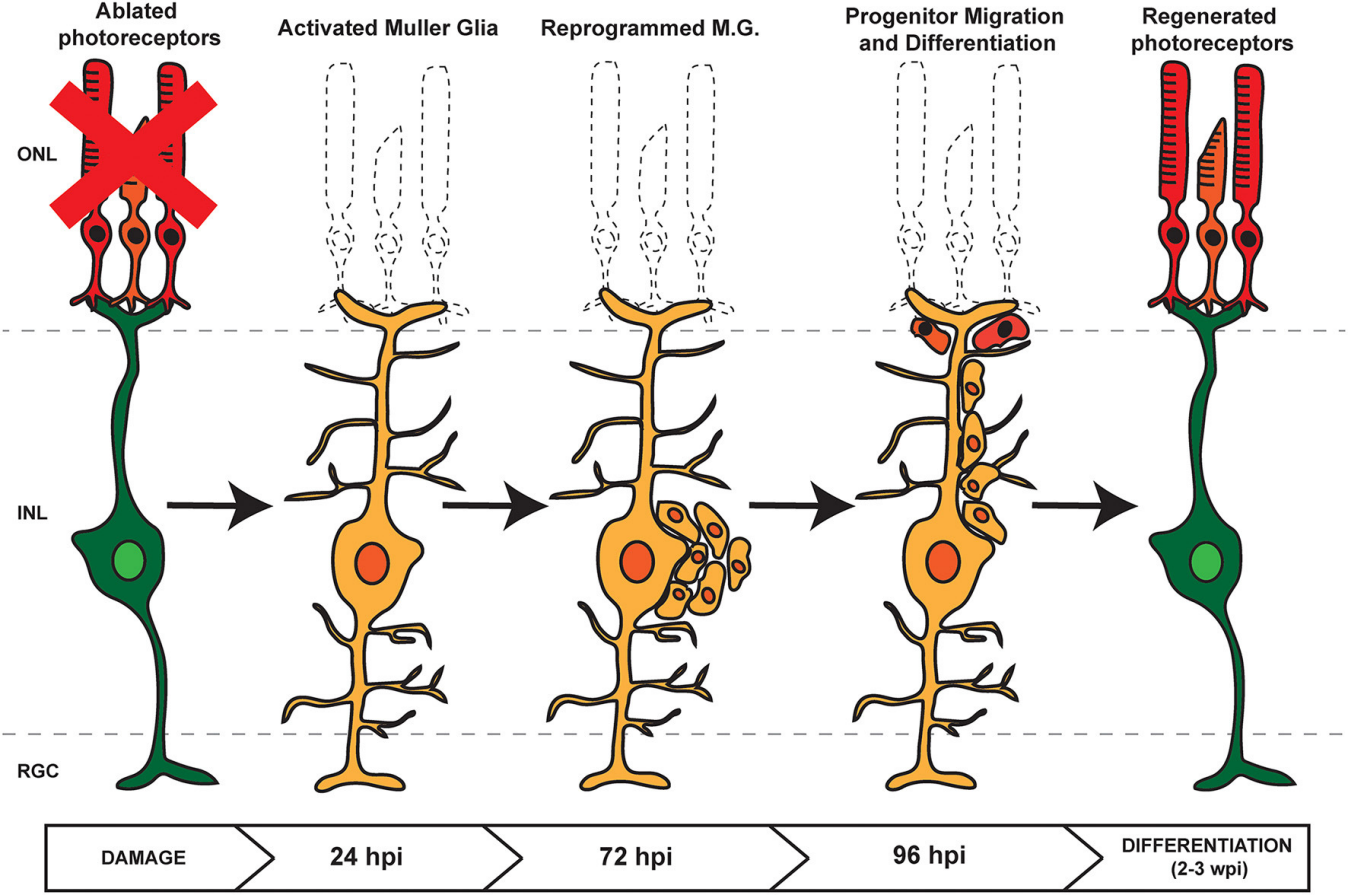
Retina Regeneration in Zebrafish. In response to retinal damage or cell loss, zebrafish Müller Glia (MG) are activated and undergo dedifferentiation. Asymmetric division allows for self renewal and the generation of proliferating retinal progenitor cells (RPCs) which cluster along MG processes. As regeneration proceeds, the RPCs migrate to the site of damage before differentiating into any lost or damaged cell types.

**Figure 2:**
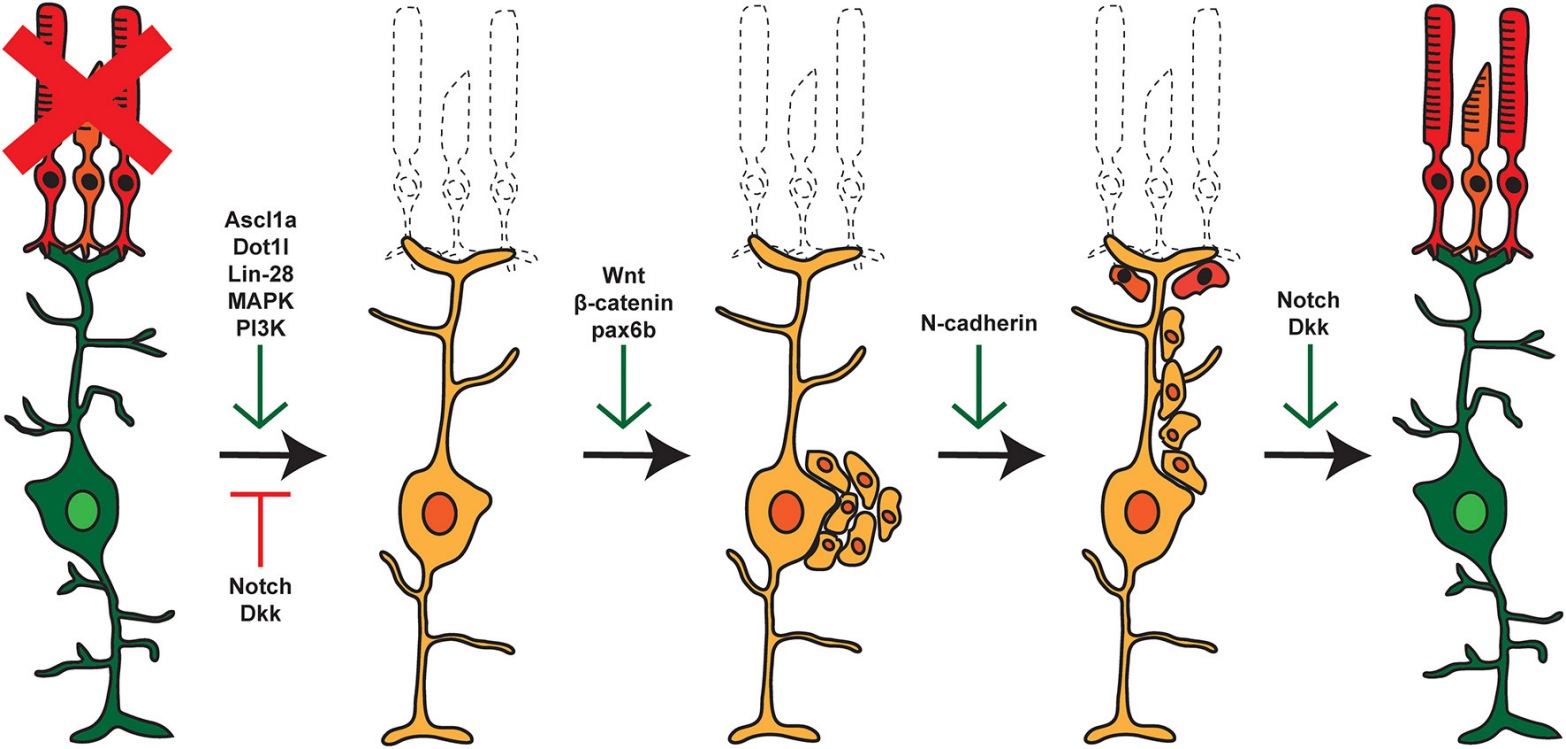
Pathways and factors involved in retina regeneration. Following damage in the zebrafish retina, multiple pathways are activated controlling dedifferentiation of MG, re-entry into the cell cycle, asymmetric cell division, generation of proliferating RPCs, and eventual differentiation into replacement cell types. Green arrows represent factors that are active at the given step, whereas red arrows represent factors that are repressed or inactive at that time.

Following damage in zebrafish, signaling cascades induce MG to dedifferentiate to a stem cell-like state and reenter the cell cycle, followed by asymmetric division for self-renewal and for the generation of proliferating RPCs (Nagashima et al., 2013). These cells cluster along MG processes and then migrate to sites of damage where they exit the cell cycle and differentiate into new cells that can replace any damaged cell type (Figure 1; Fausett and Goldman, 2006; Bernardos et al., 2007; Thummel et al., 2008b, 2010; Montgomery et al., 2010; Ramachandran et al., 2010; Qin et al., 2011; Powell et al., 2012; Taylor et al., 2012). Though the current understanding of retina regeneration is ongoing, a number of factors have been identified that transition the retina through the various stages of MG-derived retina regeneration (Wan and Goldman, 2016; Lahne et al., 2020; Figure 2). Here, we will focus on the identification and role of miRNAs during MG reprogramming and retina regeneration.

## miRNAs

miRNAs are highly conserved ~22 nucleotide (nt) RNAs that post-transcriptionally regulate gene expression (Krol et al., 2010; Bartel, 2018; Gebert and MacRae, 2019). Primary miRNA transcripts are initially processed in the nucleus into ~70 nt precursor structures by a multi-protein complex referred to as the Microprocessor, the main component of which is Drosha (Kim, 2005). After export from the nucleus, cytoplasmic processing is accomplished by another multi-protein complex that includes the enzyme Dicer, which yields ~22 nt double stranded RNAs. One of the strands is subsequently assembled into an RNA Induced Silencing Complex (RISC) (Schwarz et al., 2003; Filipowicz, 2005) containing one or more members of the Argonaute protein family (Peters and Meister, 2007). miRNA-mediated gene silencing occurs by pairing between miRNAs and their target mRNAs, usually in the 3′ UTR. Once paired, miRNAs inhibit translation and induce deadenylation leading to mRNA degradation (Giraldez et al., 2006; Guo et al., 2010).

## miRNAs and Retina Regeneration

**Figure 3:**
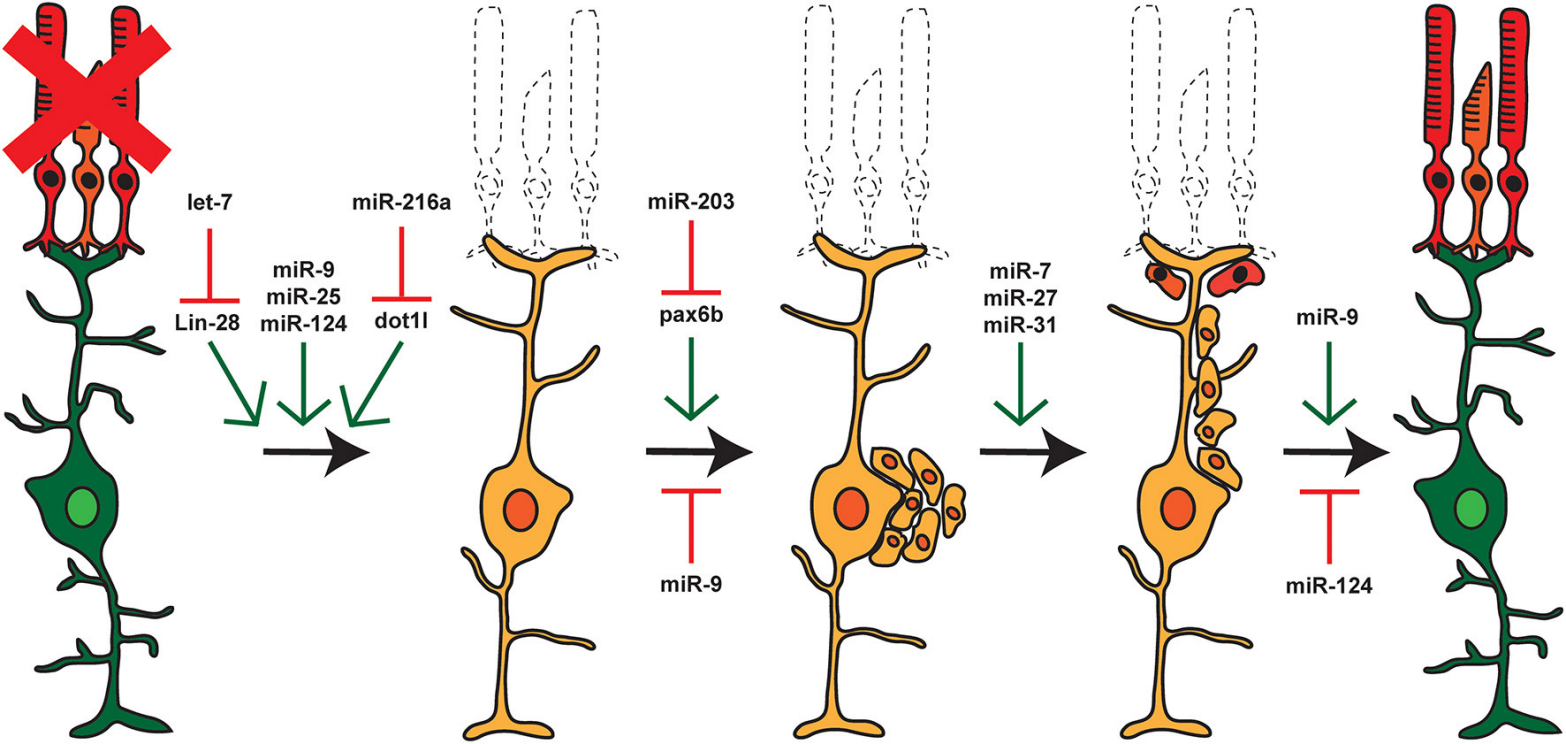
miRNAs and Retina Regeneration. The adult zebrafish retina expresses ~200 miRNAs but only a small subset of these miRNAs are differentially expressed during regeneration. RNAseq and other analyses after damage or during distinct stages of regeneration have identified miRNAs and select target mRNAs, as indicated. Green arrows represent miRNAs that are expressed or active at that given step, and red arrows represent miRNAs that are not expressed or inactive.

miRNAs were first discovered in *C. elegans* where they control development (Lee et al., 1993; Wightman et al., 1993), but they have now been shown to play important roles in a number of biological processes including metabolism, cancer, metastasis, and regeneration (Alvarez-Garcia and Miska, 2005). miRNAs have been implicated in regeneration in a number of biological models ranging from planaria to mice (Yin and Poss, 2008; Williams et al., 2009; Thatcher and Patton, 2010) and in zebrafish have been shown to regulate regeneration of the heart, fin, muscle, liver, lens, and inner ear hair cells (Tsonis et al., 2007; Liu et al., 2008, 2012; Thatcher et al., 2008; Yin et al., 2008; Song et al., 2010). Knockdown of Dicer in the adult zebrafish retina prior to constant intense light damage reduced the ability of MG to produce proliferating RPCs in response to damage (Rajaram et al., 2014a). This indicated a general requirement for miRNAs during retina regeneration. After damage, most miRNA expression levels remain unchanged or undergo only small changes during regeneration. However, specific subsets of zebrafish miRNAs show both up- and down-regulation throughout the regenerative process (Figure 3; Rajaram et al., 2014a). Similarly, a small subset of miRNAs have been implicated in controlling the reprogramming of mammalian Müller glia (Wohl and Reh, 2016b; Wohl et al., 2019).

## *let-7*/LIN-28

One of the first demonstrations of a role for miRNA involvement in retina regeneration was by the Goldman lab focusing on *let-7* (Ramachandran et al., 2010). Using a puncture damage model in zebrafish, they used a candidate gene approach to identify pluripotency factors whose expression changes during retina regeneration. One of those factors was Lin-28, an RNA-binding protein that was first discovered to regulate development in *C. elegans* as part of a double negative feedback loop with *let-7* (Moss et al., 1997; Reinhart et al., 2000). After retina damage, Lin-28 is induced downstream of the transcription factor Ascl1 and knockdown of Lin-28 inhibits retina regeneration (Ramachandran et al., 2010; Zhao et al., 2017). Lin-28 activation leads to the repression of *let-7* expression which in turn derepresses the expression of multiple regeneration and pluripotency factors including Ascl1a (Ramachandran et al., 2010). *Let-7* has 12 family members; Lin-28 inhibits the production of most *let-7* family members by recruiting a uridylyl transferase to *pre-let-7* transcripts leading to uridylation, inhibition of processing, and subsequent decay (Hagan et al., 2009; Heo et al., 2009). For MG, it appears that expression of *let-7* maintains the differentiated state, but that induction of Lin-28 after injury allows dedifferentiation by reducing *let-7* levels. This is consistent with a role for regulation of Lin-28 by *let-7* during development as well (La Torre et al., 2013; Fairchild et al., 2019).

## miRNA-203/Pax6b

In a screen to identify differentially expressed miRNAs during zebrafish MG-derived retina regeneration, *miR-203* was found to be downregulated and that artificially maintaining its expression blocked retina regeneration (Rajaram et al., 2014b). Previously, *miR-203* downregulation had been shown to be required for caudal fin regeneration and it is similarly downregulated during mouse skin regeneration (Lena et al., 2008; Thatcher et al., 2008; Yi et al., 2008). These data supported a role for *miR-203* in promoting differentiation and repressing stemness. Elevated levels of *miR-203* inhibit proliferation of RPCs and are essential for the formation of clusters of RPCs that are commonly observed along MG processes (Figure 1; Rajaram et al., 2014b). However, *miR-203* does not play a role in dedifferentiation of MG during earlier stages of regeneration, indicating a temporal expression pattern throughout the regenerative processes.

In a search for potential mRNA targets for *miR-203*, bioinformatic and reporter analyses identified the Paired-box gene 6b (Pax6b) (Rajaram et al., 2014b). Pax6 expression is essential for eye development across species (Shaham et al., 2012; Baker et al., 2018). There are two Pax6 paralogs in zebrafish (Pax6a and Pax6b) with distinct roles during retina regeneration (Thummel et al., 2010). Misregulation or loss of Pax6 expression can lead to multiple mammalian visual system defects, most commonly aniridia or nystagmus (Lima Cunha et al., 2019). Consistent with a role for *miR-203* controlling progenitor proliferation and cluster formation, the Hyde lab had previously shown that *pax6b* in zebrafish is expressed in NPCs and is required for the formation of clusters (Thummel et al., 2008a). *miR-203* must be repressed during regeneration to allow for *pax6b* expression and the formation of RPC clusters on MG.

## miR-9/miR-124/PTB/nPTB

The Fu laboratory discovered that repression of the hnRNP protein Polypyrimidine tract-binding protein 1 (PTB1) can convert fibroblasts into a neuronal cell type fate (Xue et al., 2013). PTB1 is a ubiquitously expressed regulator of alternative splicing that itself undergoes alternative splicing to autoregulate its levels (Wollerton et al., 2004). A neuronal paralog of PTB (PTB2 or nPTB) controls multiple neuronal alternative splicing events and its levels are regulated by an alternative splicing event controlled by PTB1 (Boutz et al., 2007; Makeyev et al., 2007). PTB1 is expressed in neuronal precursor cells and glia; nPTB is expressed during neuronal induction and maturation, with the expression of both paralogs decreasing during neuronal differentiation (Boutz et al., 2007; Hu et al., 2018). Two regulatory loops control PTB and nPTB expression through the action of *miR-9* and *miR-124*, respectively, along with the transcription factors REST and BRN2 (Makeyev et al., 2007; Hu et al., 2018). Intriguingly, it was recently shown that targeted destruction of mRNAs encoding PTB in the mouse retina can cause MG to dedifferentiate (Zhou et al., 2020). Using an NMDA damage model in mice, targeting of mRNAs encoding PTB by CRISPR/CasRx led to MG dedifferentiation and replacement of damaged ganglion cells. Also, targeted depletion of mRNAs encoding PTB by antisense oligonucleotides led to the conversion of astrocytes to dopaminergic neurons (Qian et al., 2020). Together, the data support the surprising finding that targeting a single, widely expressed regulator of alternative splicing can drive the conversion of glia to neurons.

Even though targeting of PTB provides an attractive single gene approach for retina regeneration, an alternative would be to deliver *miR-9* and *miR-124* to not only regulate PTB and nPTB expression, but also to upregulate the transcription factors REST and BRN2 (Hu et al., 2018). It will be crucial to determine whether the expression of PTB and nPTB are controlled in the retina by the *miR-9 and miR-124* regulatory loops. If so, regulation of REST by these regulatory loops could control expression of NeuroD which plays a role in neuronal cell fate, and Ascl1 which is required for retina regeneration (Ramachandran et al., 2010; Cherry et al., 2011). Similarly, regulation of BRN2 could in turn control expression of neuronal maturation genes including NEUN and NLGN2. Gene profiling experiments did not observe significant changes in PTB between control and neurogenic MG (Hoang et al., 2020) so it may be that increased expression or delivery of *miR-9* and *miR-124* mimics might drive broader overall gene expression changes to induce retina regeneration rather than just targeting PTB (Wohl and Reh, 2016b).

## miR-9/miR-124/Ascl1

The Reh lab used dissociated mouse MG cultures to identify factors and miRNAs that can stimulate reprogramming of retinal cell fate (Pollak et al., 2013; Wohl and Reh, 2016b). Induced overexpression of the transcription factor Asc1 can reprogram mouse MG into neurogenic RPCs and the effects of Ascl1 overexpression can be augmented by parallel overexpression of both *miR-124* and *miR-9* (Wohl and Reh, 2016b). This agrees nicely with the regulatory loops controlling neuronal induction and maturation controlled by *miR-124* and *miR-9* but to date, whether these loops control gene expression in the retina is not clear (Hu et al., 2018).

## miR-25/*let-7*/miR-124/miR-9

In a follow up study to the effects of *miR-9* and *miR-124* on reprogramming of mammalian MG, the Reh lab profiled miRNA expression patterns in sorted mouse MG and RPCs, and also utilized a conditional mouse model with a MG-specific deletion of Dicer (Wohl and Reh, 2016a; Wohl et al., 2017, 2019). After Dicer knockdown, the most significantly altered gene was Brevican (BCAN) which is targeted by *miR-9*. More broadly, loss of Dicer led to a dramatic loss of retinal architecture indicating an important role for miRNAs in the maintenance of homeostasis, and also supporting the overall importance of miRNAs in regeneration, a process which is blocked by the loss of Dicer (Rajaram et al., 2014a). When comparing sorted MG and RPCs, Ascl1 expression was found to be enhanced by either overexpression of *miR-25* and *miR-124* or by downregulation of *let-7* (Wohl et al., 2019). Targeting of Lin-28 by *let-7* can explain indirect regulation of Ascl1.

*miR-25* is expressed as part of the highly conserved *miR-106b/25* cluster with roles in DNA damage response, cell cycle regulation, cell proliferation, migration, and differentiation (Sarkozy et al., 2018). Additional potential target mRNAs for *miR-25* include REST, Tpm1, Itgb1, Ctdsp1, Rcor1, and Ccnd2 (Sarkozy et al., 2018; Wohl et al., 2019). Besides targeting the protein components of the REST complex, another key predicted target of *miR-25* (and *let-7*) is the Wnt inhibitor Dickkopf 3 (Dkk3) (Huo et al., 2016; Wohl et al., 2019). This is consistent with the requirement for Wnt activation during retina regeneration in zebrafish and possibly mice (Osakada et al., 2007; Ramachandran et al., 2011; Kara et al., 2019).

*miR-124* is one of the most abundant miRNAs in the adult brain and is thought to be a master regulator of neuronal differentiation, including its role in regulating PTB expression (Yeom et al., 2018) and targeting of REST (Wohl et al., 2019). *miR-124* is known to reduce the expression of a small phosphatase specific for phosphoserines in the C-terminus of RNA Polymerase II called SCP1, which is a repressor of neuron-specific transcription in nonneuronal cells and is also a component of REST (Cao et al., 2007; Makeyev et al., 2007; Visvanathan et al., 2007).

## miR-216a/Dot1l

*miR-216a* is another well-known miRNA that plays a role in gliogenesis during retinal development by indirectly regulating Notch signaling (Olena et al., 2015). For MG-derived regeneration, *miR-216a* can be thought of as a gatekeeper miRNA in reprogramming events, as its expression holds MG in a quiescent state until the retina is damaged (Kara et al., 2019). One mechanism for how *miR-216a* can serve as a gatekeeper controlling the early steps of regeneration is by targeting mRNAs encoding the Disruptor of telomeric silencing 1-like (DOT1l) gene (Kara et al., 2019). Dot1l plays a role in many chromatin-associated functions such as gene-transcription, heterochromatin formation, and DNA repair, as well as the response to DNA damage and chemotherapy responsiveness (McLean et al., 2014). After retinal damage in zebrafish, *miR-216a* is downregulated allowing increased expression of Dot1l which leads to activation of Wnt target genes, presumably by altering chromatin accessibility surrounding these genes (Kara et al., 2019). The idea that dedifferentiation of MG involves changes in chromatin accessibility is expected and has been experimentally supported (Jorstad et al., 2017; Mitra et al., 2018; VandenBosch et al., 2020). When combined with Ascl1 overexpression in an NMDA damage model in adult mice, the addition of the general histone deactylase inhibitor trichostatin A (TSA) stimulated neuronal regeneration which was otherwise only observed in developing mice <12 days old (Jorstad et al., 2017). Also, after puncture damage in zebrafish, inhibition of histone deacetylases by valproic acid suppressed the formation of MG-derived NPCs (Mitra et al., 2018). Further work is needed to identify specific genes whose chromatin accessibility changes during MG dedifferentiation and eventual re-differentiation, but miRNA control is an attractive regulatory mechanism that might allow for fine-tuned control of signaling cascades that are induced after cellular damage.

## miR-7/miR-27/miR-31

In a screen to identify differentially expressed miRNAs during zebrafish retina regeneration, *miR-7, miR-27, and miR-31* were all found to be upregulated at 72 h post light damage and targeted knockdown of these miRNAs led to decreased numbers of proliferating cells (Rajaram et al., 2014a). The timing of overexpression and the effects of loss of function of these miRNAs during regeneration suggest that they function during continued RPC proliferation and migration, similar to the proposed role for Pax6a (Thummel et al., 2010; Rajaram et al., 2014b). Exact targets for these miRNAs remain to be identified but related experiments summarized below might provide hints to possible mRNA targets for these miRNAs.

*miR-7* regulates multiple signaling pathways including epidermal growth factor receptor (EGFR), insulin-like growth factor (IGF), Hedgehog, Notch, and the mammalian target of rapamycin (mTOR) pathways, as well as being a key regulator of *pax6a* in mice (Needhamsen et al., 2014; Baba et al., 2015; Zhao et al., 2015). In the forebrain, *miR-7* regulates *pax6* to spatially control the origin of dopaminergic neurons (de Chevigny et al., 2012)_._

*miR-27* promotes blood vessel development, particularly in the eye (Liu et al., 2020). *miR-27* also plays an important role in mitochondrial dynamics as it inhibits degradation of damaged mitochondria by regulating PINK1 and also by inhibiting mitochondrial fission factor (MFF) expression, which increases mitochondrial membrane potential (Tak et al., 2014; Kim et al., 2016). Loss of *miR-27c* in the retina decreases proliferation of MG-derived RPCs during regeneration, a similar phenotype to what occurs with loss of *miR-27a and miR-27b* in muscle progenitor cell proliferation (Crist et al., 2009; Lozano-Velasco et al., 2011; Rajaram et al., 2014a).

*miR-31* is a well-studied miRNA with a major target being transcripts encoding the myogenic determining factor Myf5 (Crist et al., 2012). *miR-31* levels affect both satellite cell differentiation *ex vivo* and muscle regeneration *in vivo*, making *miR-31* a miRNA of great interest in regard to stem cell research. *miR-31* is a regulator of many signaling pathways relevant to developmental biology and cancer including the Prlr/Stat5, TGFβ, and Wnt/β-catenin pathways (Lv et al., 2017). Additionally, *miR-31* has been shown to coordinate signals from BMP, TGFβ, and Wnt pathways in intestinal stem cells to regulate their proliferation, regeneration, and homeostasis, further reinforcing its impact in progenitor proliferation during regeneration (Tian et al., 2017).

## Discussion

miRNAs regulate gene expression by binding to 3′ UTR elements leading to deadenylation and subsequent degradation of mRNA targets (Giraldez et al., 2006; Guo et al., 2010). Target recognition typically involves imperfect base pairing, often within the seed region (nucleotides 2-8) that is commonly used to predict miRNA targets (Li et al., 2008; Broughton et al., 2016; Bartel, 2018). Because the base pairing interaction is imperfect, miRNA target prediction algorithms can identify candidate mRNAs, but experimental validation is necessary to confirm direct silencing. Thus, for all of the miRNAs discussed above, there are likely additional mRNA targets that could affect the same processes, ranging from regeneration to signaling cascades. Further work is required to identify the complete set of miRNAs that regulate retina regeneration and the target genes they control.

Because miRNAs are largely conserved among vertebrates, the expection is that discoveries across species will illustrate general principles and uncover common mechanisms. Fortunately, it does not appear that there are hundreds of miRNAs that regulate MG reprogramming and, for the subset that has been identified, they comprise an attractive class of regulatory molecules, especially because retinal architecture and MG gene expression patterns are evolutionarily conserved suggesting that elucidating overall gene regulation will help to understand the inability of mammalian MG to initiate regeneration. It remains possible that species-specific networks or species-specific factors might control the passage of MG from quiescence to reactivity and further to the generation of proliferating RPCs, but the weight of evidence thus far seems to suggest that activating MG-derived regeneration cascades in mammals will be possible by derepression of existing pathways as opposed to delivery of species-specific genes (Ahmad et al., 2011; Lust and Wittbrodt, 2018; Hoang et al., 2020).

## Therapeutic miRNA

The accessibility of the eye and the small size of miRNAs raises the possibility of delivering miRNA mimics or antisense RNAs (antagomirs) that block miRNA function for therapeutic purposes. The challenges for such experiments are at least three-fold: (1) how to target injected miRNAs to MG; (2) whether single injections will be sufficient to induce a regenerative response; and (3), avoidance of off-target effects if high concentrations are required. While direct injections of miRNAs or antagomirs are possible, the discovery that extracellular vesicles (EVs) can be used to deliver therapeutic cargo opens an exciting possibility for cell-specific delivery (Mead and Tomarev, 2020). Recently, it has become increasingly clear that miRNAs can engage in cell-cell signaling via EVs (Maas et al., 2017; O'Brien et al., 2020). Transfer of miRNAs or other cargo by EVs might play a role in patterning the retina during development and may also be a key part of degeneration and regeneration (Bian et al., 2020). Indeed, retina regeneration can be induced by delivery of EVs (Didiano et al., 2020). Although EVs were shown to induce the early stages of retina regeneration, the effects were quite modest. However, as a therapeutic tool, it may be possible to load EVs with specific miRNAs or other small molecules for delivery to MG after intravitreal or subretinal injection. The miRNAs described in this review may be candidate miRNAs for the development of designer EVs that could be targeted to MG to induce retina regeneration.

## Author Contributions

GK made the figures. All authors contributed to the writing.

## Conflict of Interest

The authors declare that the research was conducted in the absence of any commercial or financial relationships that could be construed as a potential conflict of interest.
